# Decreasing the expression of PICALM reduces endocytosis and the activity of β-secretase: implications for Alzheimer’s disease

**DOI:** 10.1186/s12868-016-0288-1

**Published:** 2016-07-18

**Authors:** Rhian S. Thomas, Alex Henson, Amy Gerrish, Lesley Jones, Julie Williams, Emma J. Kidd

**Affiliations:** School of Pharmacy and Pharmaceutical Sciences, Cardiff University, Redwood Building, King Edward VII Avenue, Cardiff, CF10 3NB UK; MRC Centre for Neuropsychiatric Genetics and Genomics, School of Medicine, Cardiff University, Hadyn Ellis Building, Maindy Road, Cardiff, CF24 4HQ UK; West Midlands Regional Genetics Laboratory, Birmingham Women’s NHS Foundation Trust, Mindelsohn Way, Edgbaston, Birmingham, B15 2TG UK

**Keywords:** Alzheimer’s disease, Amyloid precursor protein, β-C-terminal fragment, β-Secretase, Clathrin-mediated endocytosis, PICALM

## Abstract

**Background:**

Polymorphisms in the gene for phosphatidylinositol binding clathrin assembly protein (PICALM), an endocytic-related protein, are associated with a small, increased risk of developing Alzheimer’s disease (AD), strongly suggesting that changes in endocytosis are involved in the aetiology of the disease. We have investigated the involvement of PICALM in the processing of amyloid precursor protein (APP) to understand how PICALM could be linked to the development of AD. We used siRNA to deplete levels of PICALM, its isoforms and clathrin heavy chain in the human brain-derived H4 neuroglioma cell line that expresses endogenous levels of APP. We then used Western blotting, ELISA and immunohistochemistry to detect intra- and extracellular protein levels of endocytic-related proteins, APP and APP metabolites including β-amyloid (Aβ). Levels of functional endocytosis were quantified using ALEXA 488-conjugated transferrin and flow cytometry as a marker of clathrin-mediated endocytosis (CME).

**Results:**

Following depletion of all the isoforms of PICALM by siRNA in H4 cells, levels of intracellular APP, intracellular β-C-terminal fragment (β-CTF) and secreted sAPPβ (APP fragments produced by β-secretase cleavage) were significantly reduced but Aβ40 was not affected. Functional endocytosis was significantly reduced after both PICALM and clathrin depletion, highlighting the importance of PICALM in this process. However, depletion of clathrin did not affect APP but did reduce β-CTF levels. PICALM depletion altered the intracellular distribution of clathrin while clathrin reduction affected the subcellular pattern of PICALM labelling. Both PICALM and clathrin depletion reduced the expression of BACE1 mRNA and PICALM siRNA reduced protein levels. Individual depletion of PICALM isoforms 1 and 2 did not affect APP levels while clathrin depletion had a differential effect on the isoforms, increasing isoform 1 while decreasing isoform 2 expression.

**Conclusions:**

The depletion of PICALM in brain-derived cells has significant effects on the processing of APP, probably by reducing CME. In particular, it affects the production of β-CTF which is increasingly considered to be an important mediator in AD independent of Aβ. Thus a decrease in PICALM expression in the brain could be beneficial to slow or prevent the development of AD.

**Electronic supplementary material:**

The online version of this article (doi:10.1186/s12868-016-0288-1) contains supplementary material, which is available to authorized users.

## Background

In 2009 Genome Wide Association Studies (GWAS) were published describing polymorphisms in a number of genes associated with an increased risk of developing Alzheimer’s disease (AD) including *PICALM* (phosphatidylinositol binding clathrin assembly protein) [1, 2]. PICALM is involved in clathrin-mediated endocytosis [3]. Subsequently, the large IGAP (International Genomics of Alzheimer’s disease Project) study identified further genes encoding the endocytic proteins *BIN1* (bridging integrator 1) and *SORL1* (sortilin-related receptor) that reached genome-wide significance [2, 4, 5]. Pathway analysis of the IGAP study [4] identified a number of biological pathways associated with AD including the regulation of endocytosis, thus emphasising the importance of this pathway in the pathogenesis of AD and suggesting areas for development of new therapies [3].

The amyloid cascade hypothesis is the leading theory to explain the aetiology of AD [6], suggesting that understanding how the production of β-amyloid (Aβ) from amyloid precursor protein (APP) is controlled should improve intervention strategies. After synthesis, some APP is transported via the secretory pathway to the cell surface and internalised by endocytosis for processing including cleavage by one of two routes [7, 8]. In the amyloidogenic pathway, β-secretase (identified as β-site APP cleaving enzyme or BACE1) cleaves APP to give two fragments, sAPPβ and C99 [9–12]. The γ-secretase complex then cleaves C99 to produce Aβ and a C-terminal fragment (CTF) [12–15]. The alternative non-amyloidogenic pathway involves cleavage of APP by α-secretase within the Aβ region to release sAPPα and precludes the formation of Aβ [16, 17]. It is thought that most amyloidogenic processing of APP occurs after endocytosis in the endocytic/lysosomal system [18]. Endocytosis is thus central to the production of Aβ as it controls where APP is localised in the cell and which enzymes it encounters [19].

Altered CME has been described previously in AD. Early endocytic changes (an increase in number and size of Rab5-positive endosomes) have been seen in post-mortem AD brains [20, 21]. Inhibition of CME in vivo in APP transgenic mice and dynamin-dependent endocytosis in vitro lowered Aβ levels [22, 23], while upregulation of endocytosis increased APP metabolism and Aβ secretion [18]. Therefore endocytosis is strongly implicated in AD pathogenic processes.

PICALM is involved in the recruitment of clathrin and other proteins to the membrane and thus regulates the formation of clathrin-coated pits and vesicles in CME [24]; though how it might be involved in endocytosis in AD remains unclear. However, subjects carrying the AD risk alleles in *PICALM* and *Clusterin* show significantly poorer episodic memory, a marker for AD onset [25]. In yeast, deletion of *PICALM* homologues protected against Aβ-induced toxicity [26]. In contrast, the presence of *PICALM* protective alleles appeared to be associated with an increase in PICALM mRNA expression in human brain [27] and to protect against senile plaque development [28]. Decreased PICALM expression has been noted in AD brains along with abnormal cleavage fragments of PICALM and the association of PICALM with neurofibrillary tangles [29]. Thus PICALM may play an important role in AD, but the mechanism by which it does this is unclear. Since the processing of APP to Aβ is dependent on CME, our study aimed to investigate the contribution of PICALM to modulating the metabolism of APP and whether endocytotic processes were involved in that modulation.

We used the human brain-derived H4 neuroglioma cell line that expresses endogenous levels of APP as we wanted to look at the effects of PICALM on physiologically relevant levels of APP, more relevant to the situation found in AD patients. We found that these cells expressed PICALM isoforms 1 and 2. Having depleted levels of PICALM by siRNA targeting all four isoforms, we showed that the levels of intracellular APP, the intracellular β-C-terminal fragment (β-CTF) and secreted sAPPβ (APP fragments produced by β-secretase cleavage) were significantly reduced but Aβ40 itself was not affected. Functional CME measured by transferrin uptake was significantly reduced after both depletion of PICALM and clathrin heavy chain (CHC). In contrast, depletion of clathrin did not affect APP levels but did reduce those of β-CTF. PICALM depletion altered the intracellular distribution of clathrin with less staining found in the perinuclear transgolgi region while clathrin depletion affected the subcellular pattern of PICALM labelling. Both PICALM and clathrin depletion reduced the expression of BACE1 mRNA and PICALM siRNA reduced protein levels. In contrast to the findings for depletion of all PICALM isoforms, individual depletion of PICALM isoforms 1 and 2 by specific siRNAs did not affect APP levels. Clathrin depletion had a more complicated differential effect on the isoforms, increasing isoform 1 protein and mRNA expression while decreasing isoform 2 protein expression but not affecting mRNA levels.

## Results

### Effects of siRNA to total PICALM on PICALM mRNA and protein expression

**Fig. 1:**
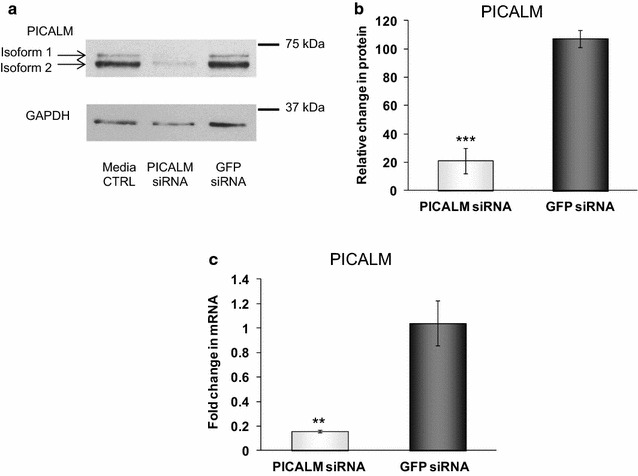
PICALM levels in H4 cells following siRNA treatments. **a** Representative immunoblot of PICALM after transfecting H4 cells for 48 h with control siRNA (GFP siRNA) or siRNA targeting PICALM (PICALM siRNA) and **b** following densitometric analysis. Data are presented as mean ± SEM as a percentage of the untreated media control after normalisation to house-keeping protein levels, n = 8, ***p < 0.001 significantly different to GFP siRNA with an unpaired Student’s *t* test. **c** PICALM mRNA expression levels using quantitative PCR, normalised to the housekeeping gene 18S and relative to the untreated cells. Data are presented as mean ± SEM, n = 6, **p < 0.01 significantly different to GFP siRNA with Mann–Whitney-U

In H4 cells, we detected two bands for PICALM using Western blotting with molecular masses of 72.2 ± 0.4 and 65.8 ± 0.9 kDa (Fig. 1a). Four main isoforms (isoforms 1–4) of PICALM have been identified [30] [NCBI RefSeq:NP_009097.2, NP_001008660.1, NP_001193875.1, and NP_001193876.1, respectively]. It is likely that the 72.2 kDa band equates to isoform 1 [NCBI RefSeq:NP_009097.2] with a predicted mass of 70.6 kDa and the 65.8 kDa band to isoform 2 [NCBI RefSeq:NP_001008660.1] with a predicted mass of 66.3 kDa. We saw no evidence of bands with a predicted mass of 69.9 kDa or 59.9 kDa, equating to isoforms 3 [NCBI RefSeq:NP_001193875.1] and 4 [NCBI RefSeq:NP_001193876.1], respectively, in our cells.

**Fig. 2:**
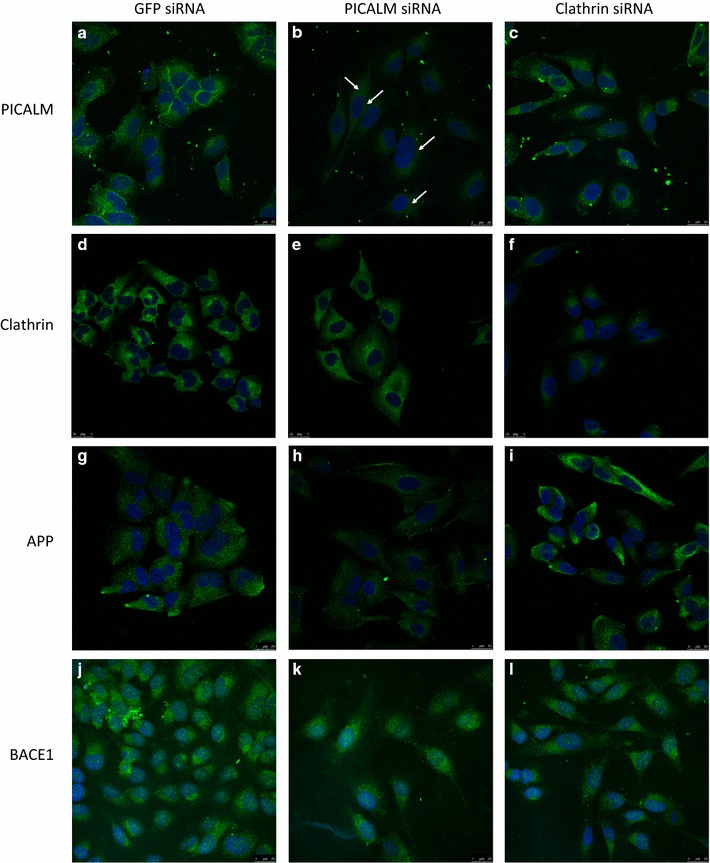
PICALM (**a**–**c**), clathrin (**d**–**f**), APP (**g**–**i**) and BACE1 (**j**–**l**) localisation after different siRNA treatments. H4 cells were incubated for 48 h with control siRNA (GFP siRNA, **a**, **d**, **g**, **j**), siRNA targeting PICALM (PICALM siRNA, **b**, **e**, **h**, **k**) or clathrin heavy chain (Clathrin siRNA, **c**, **f**, **i**, **l**). Proteins were detected using a biotinylated secondary antibody and an FITC-conjugated avidin construct (*green*), nuclei are counterstained with DAPI (*blue*). Images were taken with a Leica SP5 confocal laser scanning microscope and are representative from n = 3 (*scale bar* 25 μm). *Arrows* indicate the reduced cytoplasmic and membrane-associated staining seen after treatment with PICALM siRNA (**a**) compared to GFP siRNA (**b**)

Using an siRNA sequence directed against all the isoforms of PICALM (Seq. A), we significantly reduced the total levels of PICALM to 17.9 ± 6.2 % of levels in control cells incubated with siRNA to GFP where PICALM levels were unaffected (Fig. 1b). In parallel, PICALM mRNA levels were reduced to 16.3 ± 1.8 % of those in GFP control siRNA cells (Fig. 1c). In immunocytochemistry, total PICALM depletion greatly reduced the marked punctate cytoplasmic and membrane-associated staining for PICALM seen in GFP control cells (Fig. 2a, b). There was no apparent perinuclear labelling seen for PICALM in GFP control cells (Fig. 2a).

siRNA to PICALM did not have any significant effect on cell viability measured using the MTS assay with absorbance levels of 98 ± 4.6 and 91.4 ± 2.3 % of the media control after treatment with siRNA to GFP and PICALM, respectively. There was also no significant difference in mean cell numbers as a percentage of the media control after treatment with siRNA targeting PICALM (56.2 ± 15.4 %) compared to mean cell numbers following treatment with siRNA targeting GFP (82.0 ± 8.2).

### Effects of siRNA to total PICALM on endocytosis

**Fig. 3:**
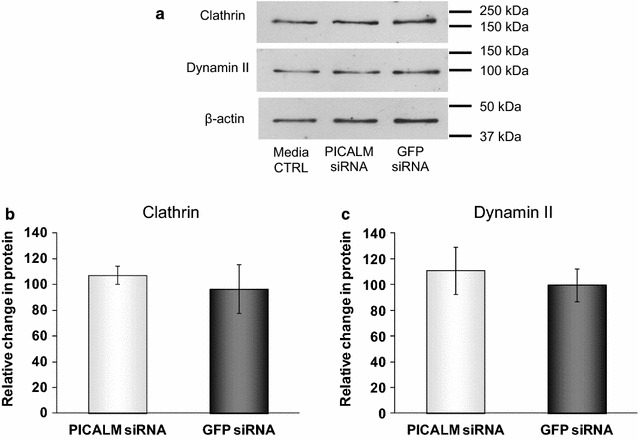
Clathrin and dynamin II protein expression levels following siRNA treatments. **a** Representative immunoblots of clathrin and dynamin II after incubating H4 cells for 48 h with media, control siRNA (GFP siRNA) or siRNA targeting PICALM (PICALM siRNA), **b** densitometric analysis of clathrin, n = 6 and **c** densitometric analysis of dynamin II, n = 4. Data are presented as mean ± SEM as a percentage of the untreated media control after normalisation to house-keeping protein levels

**Fig. 4:**
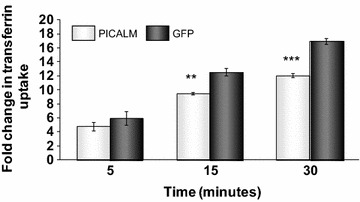
Effect of PICALM siRNA on clathrin-mediated endocytosis. H4 cells were incubated with either control siRNA (GFP siRNA) or siRNA targeting PICALM (PICALM siRNA) prior to incubation with 100 nM Alexa488-Tf for 5, 15 or 30 minutes then trypsinised and analysed by flow cytometry. Mean fluorescent intensity data for the uptake of Alexa488-Tf are expressed as fold change over untreated control cells (0 min Alexa488-Tf) and are mean ± SEM, ***p < 0.001, **p < 0.01 significantly different to GFP siRNA with an unpaired Student’s *t* test, n = 3

Having demonstrated an extensive reduction in the levels of PICALM, we investigated whether other proteins involved in CME were affected by siRNA to PICALM. Figure 3 shows that neither levels of CHC nor dynamin II were significantly affected by the reduction in PICALM levels. In addition, siRNA to PICALM had no significant effect on CHC mRNA with a reduction in mRNA levels for CHC to 98.2 ± 17.5 % of those in GFP control siRNA cells (data not shown). However, although clathrin levels overall were unaltered, we did see a reduction in the labelling for clathrin in the perinuclear region of cells depleted of PICALM compared to control cells, probably representing the transgolgi network (Fig. 2d, e). Furthermore, depletion of total PICALM significantly reduced the uptake of transferrin to 75.9 ± 2.7 and 71.2 ± 1.7 % of GFP levels after 15 and 30 min, respectively (Fig. 4).

### Effects of siRNA to total PICALM on the metabolism of APP

**Fig. 5:**
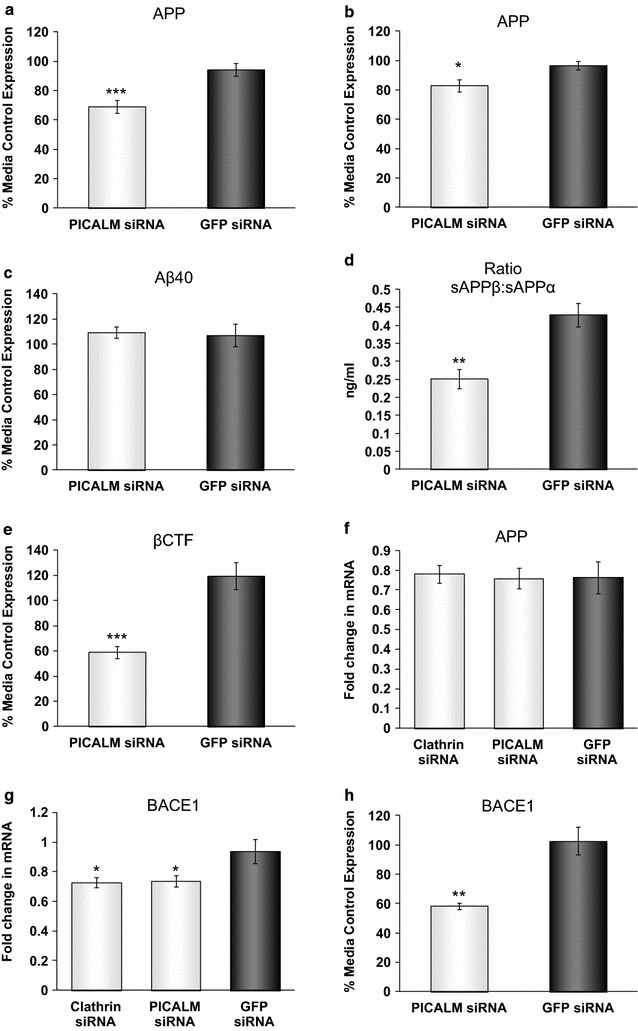
Effects of PICALM and clathrin siRNA on APP processing. H4 cells were incubated with media, control siRNA (GFP siRNA), siRNA targeting PICALM (PICALM siRNA sequence A or B) or clathrin heavy chain (Clathrin siRNA) for 48 h. Levels of APP after treatment with (**a**, PICALM siRNA Seq A) and (**b**, PICALM siRNA Seq B), **c** Aβ40, **d** sAPPβ:sAPPα, **e** βCTF and **h** BACE1 as detected by ELISA. Data are expressed as % of media control (**a**–**c**, **e**, **h**) and are mean ± SEM, *p < 0.05, **p < 0.01, ***p < 0.001 significantly different to GFP siRNA with an unpaired Student’s *t* test, n = 11 (**a**), n = 4 (**b**), n = 5 (**c**) n = 4 (**d**), n = 7 (**e**), n = 4 (**h**). **f** APP and **g** BACE1 mRNA expression levels measured using quantitative PCR, normalised to the housekeeping gene POLR2A and relative to the untreated cells. Data are expressed as mean ± SEM, *p < 0.05 significantly different to GFP siRNA with one-way analysis of variance and Dunnett’s multiple comparison test n = 6. Unless specified, cells were treated with siRNA targeting PICALM sequence A

siRNA to total PICALM significantly reduced the expression of APP to 73.3 ± 3.4 % of the GFP control levels in H4 cells (Fig. 5a). We repeated the experiment looking at APP levels using a different, but equally effective, siRNA to PICALM (Seq. B) which reduced PICALM levels to 8.2 ± 4.4 % of GFP control levels. This siRNA had a similar effect on APP expression, reducing it to 85.9 ± 4.2 % of GFP levels (Fig. 5b). Reduced levels of APP protein after PICALM siRNA were also seen by immunocytochemistry in H4 cells but the distribution of intracellular APP did not appear to be affected (Fig. 2g, h). However, the levels of Aβ40 were not affected by PICALM depletion (Fig. 5c). Aβ42 could not be measured in the H4 cells as the levels were below the detection limits of ELISAs (data not shown). Further investigation of the fragments of APP found that sAPPα was unaffected by reducing PICALM expression (data not shown). In contrast, the proportion of sAPPβ to sAPPα (expressed as a percentage) was significantly reduced by total PICALM siRNA to 59.5 ± 7.2 % of GFP control levels (Fig. 5d). βCTF levels were significantly reduced to 52.7 ± 7.8 % of GFP levels (Fig. 5e). In contrast to the effect on protein expression, mRNA levels for APP were unaffected by treatment with siRNA to PICALM compared to GFP (Fig. 5f). However, PICALM siRNA significantly reduced the amount of BACE1 mRNA compared to GFP siRNA to 80.5 ± 6.3 % (Fig. 5g). There was also a significant decrease in BACE1 protein levels to 57.6 ± 2.9 % of GFP levels as measured by ELISA following treatment with PICALM siRNA (Fig. 5h). However, no apparent differences were detectable in the amount or distribution of staining for BACE1 after PICALM depletion (β-secretase, Fig. 2j, k).

### Association of PICALM GWAS SNPs with PICALM isoform expression

Using the Gibbs et al. [31] eQTL dataset (150 healthy brains) and the UK Brain Expression Consortium eQTL dataset [32] (134 healthy brains) we found that the risk (major, G) allele of the most significant SNP in *PICALM* rs3851179 [2], and the risk allele (major, G) of the most significant SNP in *PICALM* from IGAP rs10792832 [4], are both associated with a significant decrease in PICALM isoform 2 mRNA expression in the frontal (rs10792832 p = 0.029; rs38581179 p = 0.022) and temporal cortex (rs10792832 p = 0.019; rs38581179 p = 0.014) and the medulla from the Trabzuni dataset [32] (rs3851179 and rs10792832 p = 0.011). However, there was no significant change in the expression of PICALM isoform 1 mRNA associated with either rs3851179 or rs10792832.

### Effects of siRNA to PICALM isoforms on PICALM mRNA and protein expression

**Fig. 6:**
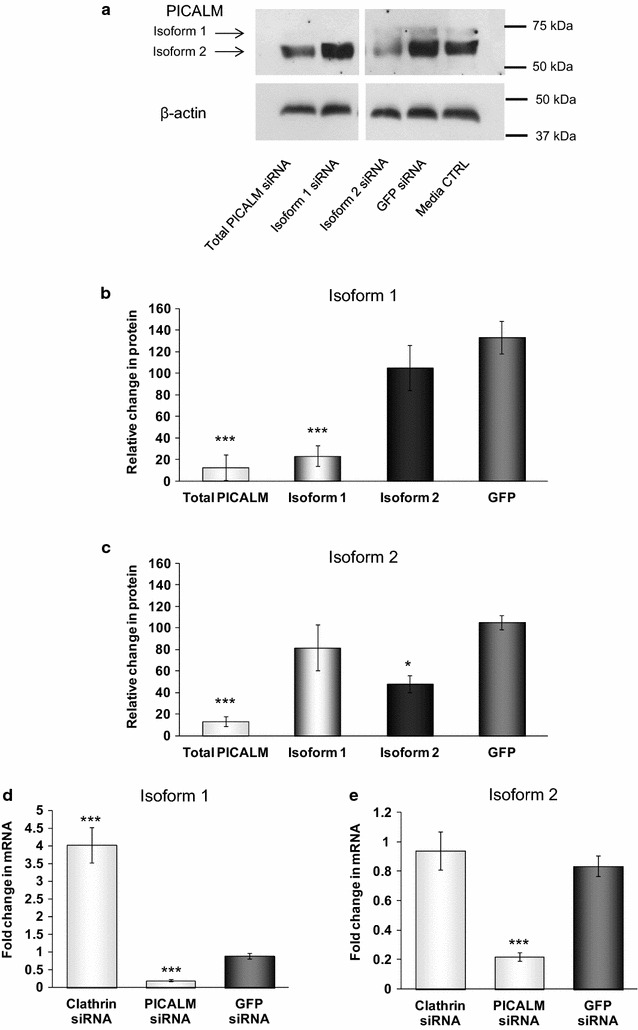
PICALM levels following siRNA treatments targeting isoforms 1 and 2 of PICALM, total PICALM and clathrin. **a** Representative immunoblots of PICALM isoforms after incubating H4 cells for 48 h with media or siRNA targeting either total PICALM (sequence A), PICALM isoform 1, PICALM isoform 2, clathrin or GFP for 48 h. **b** Densitometric Western blot analysis of the levels of PICALM, isoform 1 and **c** isoform 2. Data are presented as a percentage of the untreated media control after normalisation to house-keeping protein levels and are mean ± SEM, n = 6–7, *p < 0.05, ***p < 0.001 significantly different to GFP siRNA following one-way analysis of variance and Dunnett’s multiple comparison test (**b**) or Kruskal–Wallis test and Dunn’s multiple comparison test (**c**). **d** PICALM isoform 1 and **e** PICALM isoform 2 mRNA expression levels measured using quantitative PCR, normalised to the housekeeping gene POLR2A and relative to the untreated cells, n = 5–6, ***p < 0.001 significantly different to GFP siRNA following one-way analysis of variance and Dunnett’s multiple comparison test

Given that the *PICALM* SNPs were associated with differential changes in the expression of the isoforms of PICALM, we examined the contribution of the isoforms of PICALM to its functions in endocytosis and potential involvement with the processing of APP. We designed siRNA to the two most abundant isoforms we identified in the H4 cells, isoforms 1 and 2. Figure 6a, b shows that an siRNA to isoform 1 significantly depleted the expression of this isoform to 19.3 ± 8.6 % of GFP levels, without significantly affecting isoform 2. It was very difficult to design an siRNA for isoform 2 which did not have a significant effect on isoform 1 because of their overlapping sequences and the most selective siRNA for isoform 2 produced a smaller significant reduction to 48.2 ± 9.0 of GFP levels, without affecting isoform 1 (Fig. 6a, c). mRNA for isoforms 1 and 2 were both significantly reduced to 20.7 ± 2.3 and 23.8 ± 1.9, respectively, from GFP levels by siRNA to total PICALM (Fig. 6d, e).

### Effects of siRNA to isoforms 1 and 2 of PICALM on the metabolism of APP

**Fig. 7:**
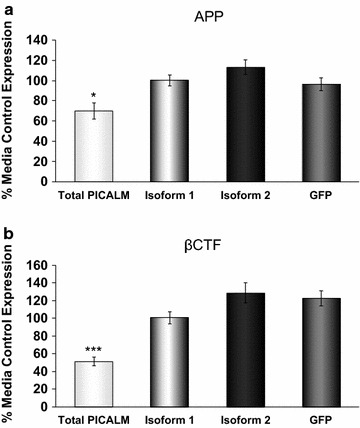
Effects of PICALM siRNA targeting isoforms 1 and 2 of PICALM on APP processing. Levels of **a** APP and **b** βCTF in H4 cells, as detected by ELISA following incubation with media, or siRNA targeting either total PICALM (sequence A), PICALM isoform 1, PICALM isoform 2 or GFP for 48 h. Data are expressed as % of media control and are mean ± SEM, *p < 0.01, ***p < 0.001 significantly different to GFP siRNA with a one-way analysis of variance and Dunnett’s multiple comparison test, n = 5 (**a**) and, n = 4–5 (**b**)

In contrast to the siRNA to total PICALM which significantly reduced APP levels, neither siRNA to isoform 1 nor isoform 2 significantly affected the levels of APP (Fig. 7a). Similarly, only the siRNA to total PICALM significantly reduced the cleavage of APP to the βCTF fragment with the siRNA to isoforms 1 and 2 having no significant effect (Fig. 7b).

### Effects of siRNA to clathrin heavy chain on endocytosis and APP expression

**Fig. 8:**
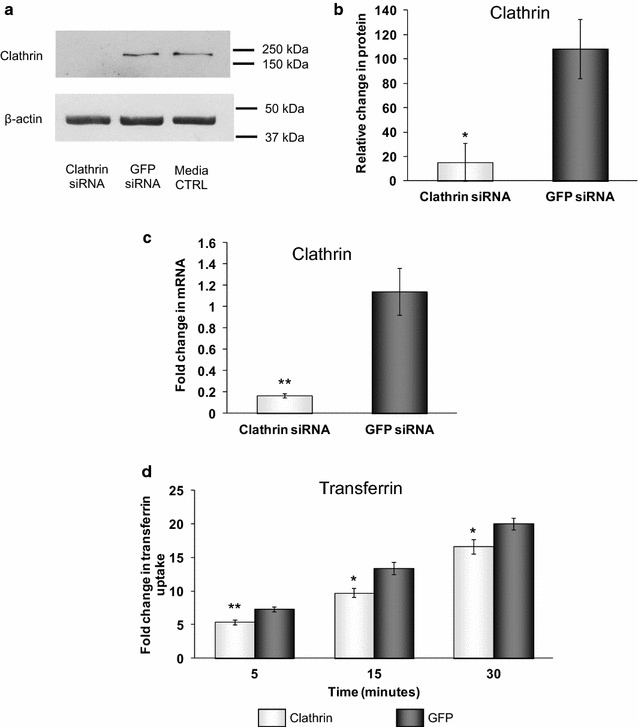
Effects of clathrin siRNA on clathrin-mediated endocytosis. H4 cells were incubated with media, control siRNA (GFP siRNA) or siRNA targeting clathrin heavy chain (Clathrin siRNA) for 48 h. **a** Representative immunoblot of clathrin expression and **b** following densitometric analysis. Data are presented as a percentage of the untreated media control after normalisation to house-keeping protein levels and are mean ± SEM, n = 3, *p < 0.05 significantly different to GFP siRNA with an unpaired Student’s *t* test. **c** Clathrin mRNA expression levels using quantitative PCR, normalised to the housekeeping gene 18S and relative to the untreated cells. Data are presented as mean ± SEM, n = 6, **p < 0.01 significantly different to GFP siRNA with Mann–Whitney-U. **d** H4 cells were incubated with 100 nM Alexa488-Tf for 5, 15 or 30, minutes then trypsinised and analysed by flow cytometry. Mean fluorescent intensity (MFI) data are expressed as fold change over untreated control cells (0 min Alexa488-Tf) and are mean ± SEM *p < 0.05,**p < 0.01 significantly different to GFP siRNA with an unpaired Student’s *t* test, n = 4–5

**Fig. 9:**
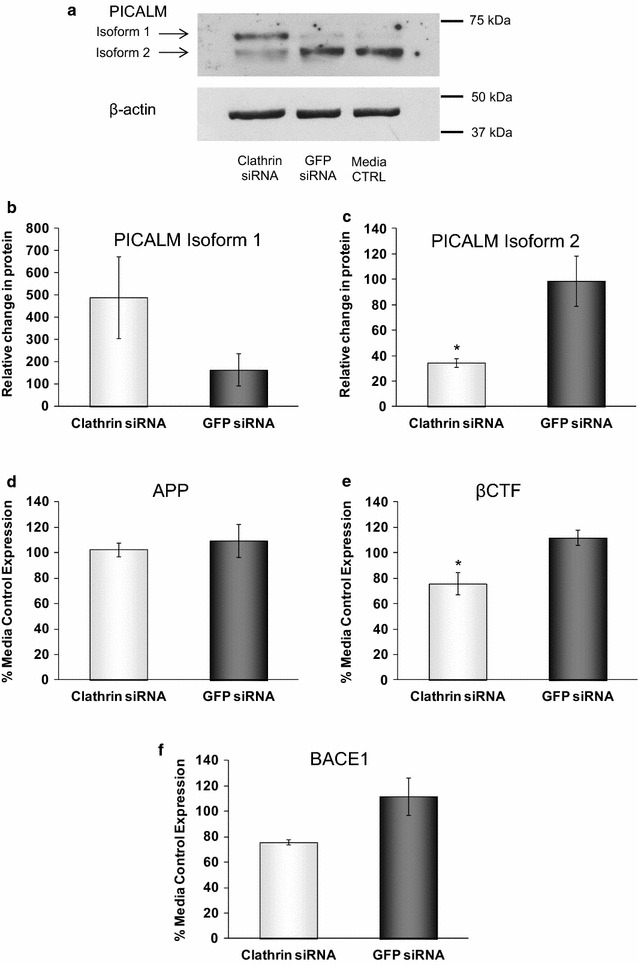
Differential effects of clathrin siRNA on isoforms 1 and 2 of PICALM and APP processing. H4 cells were incubated with media, control siRNA (GFP siRNA) or siRNA targeting clathrin heavy chain (Clathrin siRNA) for 48 h. **a** Representative immunoblot of isoforms 1 and 2 of PICALM and **b** following densitometric analysis of isoform 1 and **c** isoform 2. Data are presented as a percentage of the untreated media control after normalisation to house-keeping protein levels and are mean ± SEM. Levels of **d** APP, **e** βCTF and **f** BACE1 as detected by ELISA. Data are expressed as % of media control and are mean ± SEM. N = 3 for all, *p < 0.05 significantly different to GFP siRNA with an unpaired Student’s *t* test

Having shown that depletion of PICALM affected functional endocytosis, clathrin localisation and APP processing, we examined whether knocking down clathrin itself would have any effect on PICALM or APP processing. Using siRNA to CHC and compared to GFP siRNA-treated control cells, we significantly reduced the expression of CHC protein to 14.9 ± 1.5 % (Fig. 8a, b), CHC mRNA to 17.3 ± 3.9 % (Fig. 8c) and greatly reduced cell staining for clathrin (Fig. 2f). This reduction in clathrin produced concomitant significant decreases in CME to 81.9 ± 7.4, 75.9 ± 2.7 and 71.2 ± 6.4 % of GFP levels after 5, 15 and 30 min, respectively (Fig. 8d). siRNA to CHC had no effect at all on the mRNA for total PICALM (data not shown). It did not significantly affect the total amount of PICALM at the protein level but did differentially affect the expression of isoforms 1 and 2 of PICALM, resulting in a significant decrease in isoform 2 expression to 38.5 ± 9.2 % of GFP control levels (Fig. 9a, c). There was also a concomitant increase in isoform 1 but this did not reach significance (Fig. 9a, b). At the mRNA level siRNA to CHC significantly increased isoform 1 mRNA to 426.4 ± 61.5 % (Fig. 6c) but had no significant effect on isoform 2 mRNA (Fig. 6d). Following siRNA to CHC, labelling for PICALM was altered with more pronounced staining seen in the cytoplasm and less at the membrane (Fig. 2c). However, unlike the effect of the decrease in PICALM on APP processing, this reduction in CHC expression did not significantly alter the levels of APP (Fig. 9d) but did significantly reduce the levels of βCTF to 67.4 ± 4.5 % of GFP levels (Fig. 9e). Similarly to PICALM siRNA, mRNA levels for APP were unaffected by CHC siRNA (Fig. 5f) but BACE1 mRNA was significantly reduced by CHC siRNA to 79.6 ± 6.0 % (Fig. 5g). BACE1 protein levels measured by ELISA were not significantly altered by CHC siRNA (Fig. 9f). The labelling for APP and BACE1 were not affected by siRNA to CHC (Fig. 2i, l).

## Discussion

We have demonstrated that reducing the expression of PICALM or clathrin inhibits CME to a similar extent and reduces β-secretase-mediated cleavage of APP but only PICALM siRNA affects the levels of APP. Thus PICALM can influence the processing of APP via more than one route possibly contributing to its role as a risk factor for AD.

The gene for PICALM gives rise to a number of isoforms [27, 33–35] of which 4 appear to be the most common [30]. In the H4 cells we consistently found isoforms 1 and 2 with apparent higher expression levels of isoform 2 but did not detect isoforms 3 or 4. Therefore all further work concentrated on isoforms 1 and 2. A similar pattern has been described by other authors with isoforms 1 and 2 being the predominant forms [24, 34–37]. Our two different siRNA sequences (Seqs. A and B), directed against a region common to all four isoforms, consistently demonstrated a large reduction in the expression of PICALM with no significant toxic effects from either measure. Thus our data suggest that the reduction was specific to PICALM and that PICALM is not critical for cell survival.

Reducing PICALM expression by siRNA significantly inhibited the uptake of transferrin by functional CME in the H4 cells suggesting that, in these cells, PICALM is essential for normal levels of CME to occur, probably due to its involvement in the formation of clathrin-coated vesicles [24, 35]. These data agree with other findings on functional CME where reducing [24, 38, 39] or increasing [35, 38] the expression of PICALM resulted in changes in transferrin uptake. Other studies have described no effect of siRNA for PICALM on transferrin uptake [37, 40, 41] but all these studies only measured endocytosis at short time intervals of 5–10 min so would not have seen effects occurring up to 30 min. Furthermore, this study used a quantitative assay in living cells unlike some other papers. It is also, however, possible that these findings could be explained by certain cells being capable of overcoming PICALM depletion by increased reliance on its homologue, AP180 [24, 33].

The consequent decrease in functional endocytosis following PICALM depletion does not involve dynamin or a change in the levels of clathrin since neither mRNA nor protein levels for CHC were altered by knockdown of PICALM. However, the localisation of clathrin in the transgolgi network was reduced by PICALM depletion possibly due to redistribution in the cell since overall protein expression was unaltered. A reduction in clathrin expression in the transgolgi network has been described previously for both decreasing [24] and increasing [35] the expression of PICALM, suggesting that PICALM is important for the correct localisation of clathrin in the cell. PICALM itself was not present in the perinuclear region, confirming previous findings [24, 35], so it is most likely to have an indirect effect on the targeting of clathrin to different subcellular areas. The control of trafficking between the early endosome and the transgolgi network involves many proteins so depletion of PICALM could affect multiple targets [42]. PICALM is very important for controlling the endocytosis of R-SNAREs (Soluble NSF Attachment Protein Receptors) such as VAMP2, 3 and 8 which are necessary for the fusion of endocytic vesicles containing various cargoes with endosomes or the plasma membrane for recycling [43]. VAMP3 is also involved with the tethering of endosomes to the transgolgi network [42] so affecting VAMP3 function by depleting PICALM could reduce the amount of clathrin in the transGolgi network. Depleting cells of clathrin using siRNA to clathrin heavy chain significantly reduced transferrin uptake as expected [44]. Interestingly, we saw a different time course for depletion of clathrin or PICALM to affect significantly the uptake of transferrin. Al Soraj and co-workers [44] found the largest effect of clathrin depletion was at early time points and suggested that, at later time points, the effect of reduced recycling of transferrin from endosomes became more important. Thus the effects of PICALM depletion may be affected less by recycling from endosomes.

Consistent with previous findings showing that APP is internalised by endocytosis [45] and that PICALM is involved in this process [41], we saw a decrease in the expression of intracellular APP after depletion of PICALM. The specificity of this effect on APP to PICALM was confirmed using a second total PICALM siRNA sequence. Xiao et al. [41] did not see an effect on full-length APP after depletion of PICALM possibly due to their use of cells overexpressing APP where normal physiological mechanisms might be affected. However, the consequence of depleting PICALM on APP levels is additional to an effect on the formation of clathrin-coated vesicles as reducing clathrin did not affect APP levels. PICALM can affect transcription [46], but the levels of APP mRNA were not altered by depleting PICALM so the change in APP is very unlikely to be due to reduced protein synthesis. It was also not due to a change in the subcellular location of APP in our cells. It is therefore more likely that this reduction in APP is due to the disruption of R-SNARE proteins by PICALM depletion. Changes in VAMP3 and VAMP 8 have been postulated to explain the increased levels of total cellular cholesterol seen after PICALM knockdown [47]. It is possible that the alterations in APP we see after PICALM siRNA could also be partly due to changes in cellular cholesterol homeostasis.

Depleting PICALM expression decreased β-secretase cleavage of APP as the levels of βCTF were reduced and a decrease in the sAPPβ/sAPPα ratio was seen. α-secretase activity was unaffected by depletion of PICALM. A role for PICALM in targeting CTF fragments of APP for degradation by autophagy has been suggested [48]. Thus an increase in β-CTF levels would be expected if PICALM levels are reduced but we saw the reverse. Therefore the decrease in β-CTF levels must occur upstream of autophagic processes, most likely via an effect on β-secretase. Although β-secretase cleavage was affected by knockdown of PICALM, the levels of Aβ40 were unaffected agreeing with other studies [39, 41, 49, 50]. βCTF is very likely to be involved in the development of the enlarged endosomes seen in both AD and Down Syndrome [51, 52]. βCTF is thought to activate rab5 via APPL1 (adaptor protein containing pleckstrin homology domain, phosphotyrosine binding domain and leucine zipper motif) [52] leading eventually to endosomal enlargement [52, 53]. βCTF may thus have an important role in the pathogenesis of AD [52, 53] and therefore decreasing PICALM expression would be protective by lowering βCTF production. The decrease in βCTF we see is likely to be due in part to the reduction in endocytosis produced by PICALM depletion decreasing the entry of APP into the cells as it was also seen after knock down of clathrin. However, the effect of PICALM depletion on βCTF was greater than that produced by clathrin, providing further support for an effect of PICALM on APP independent of CME.

Previous work has shown that PICALM affects the endocytosis of γ-secretase and this was proposed to explain the effects of depletion of PICALM on Aβ generation [49]. However, we have clearly demonstrated here an effect on the cleavage of APP by β-secretase after depletion of PICALM probably due to less APP being endocytosed into early endosomes to encounter β-secretase. There was no apparent effect on the distribution of BACE1 in PICALM-depleted cells compared to control cells. Both PICALM and clathrin depletion reduced the levels of BACE mRNA by about 20 % and PICALM depletion reduced protein levels by 44.45 % with a trend for a decrease of 39.5 % seen with CHC siRNA, suggesting an effect on BACE synthesis. The reduction in BACE1 levels is unlikely to be due to an effect on endocytosis as it was seen at the mRNA level and clathrin siRNA, which reduced functional endocytosis, did not affect BACE1 protein expression. However, this reduction in BACE1 levels is unlikely to explain the 35–60 % decrease in βCTF production obtained with PICALM and CHC depletion as a 50 % decrease in BACE1 levels in transgenic BACE1 ± mice resulted in reductions in Aβ of no more than 12–40 % [54, 55]. Therefore it appears unlikely that a change in the levels of β-secretase itself is responsible for the effects of PICALM and clathrin depletion on βCTF levels.

Having examined the effect of depletion of total PICALM on APP metabolism and, given that the expression of isoform 1 was lower than isoform 2 in H4 cells, we investigated whether the isoforms had differential effects on APP endocytosis and processing. We depleted isoform 1 and 2 individually using specific siRNAs which did not significantly affect the other isoform thus confirming that the two bands we saw in the H4 cells were indeed isoform 1 and 2. Interestingly, depletion of either isoform alone had no significant effect on the levels of APP or βCTF so both PICALM isoforms must be involved in functional endocytosis in the H4 cells. However, the differential effect of clathrin depletion on the expression of the isoforms at both the mRNA and protein level does suggest that they may have different roles in CME. Clathrin depletion increased both the protein and mRNA for isoform 1 but decreased isoform 2 protein without altering the mRNA. The discrepancy for isoform 2 may be due to differences in mRNA stability between the two isoforms. The increase in one PICALM isoform and the decrease in the other may explain why clathrin depletion had no effect on APP levels. The intracellular localisation of total PICALM was altered by clathrin depletion which could reflect differential changes in the expression of isoforms 1 and 2. We could not investigate this finding further to understand the mechanism involved as there are no commercial antibodies available which can distinguish between isoforms 1 and 2. Our findings from the eQTL data sets, showing an association between a decrease in isoform 2 mRNA expression but no effects on isoform 1 mRNA for the Harold and co-workers [2] and IGAP [4] SNPs, are interesting given that we and others find that control cells generally have higher levels of isoform 2 than isoform 1. These data suggest that the ratio of the two isoforms may be important physiologically. However, given that we found that both isoforms needed to be knocked down to decrease APP and βCTF expression, it is currently unclear how the differential association of the GWAS PICALM SNPs with isoform expression are related to the processing of APP. Our data suggest that the relationship between PICALM and APP processing is likely to be highly complex and requires further investigation. There are very few published studies examining the biological effects of manipulating the isoforms of PICALM. Kanatsu and colleagues [39] looked at the effects of overexpressing isoforms 1 and 2 on Aβ secretion but did not see any differences. Other workers have shown reversal of the protective effect of depletion of PICALM yeast homologue isoforms 1 and 2 by mammalian isoform 2 (mammalian isoform 1 expression was toxic in yeast) [26]. Parikh et al. [27] examined the association of mRNAs for various splice variants of PICALM with a SNP encoding a polymorphism in the gene associated with AD and found increased total PICALM expression correlated with the protective allele but there was no significant correlation with the splice variants.

## Conclusions

In conclusion, we have demonstrated that the depletion of PICALM in H4 cells has significant effects on the processing of APP, probably by reducing cleavage by β-secretase. The reduction in βCTF production is particularly interesting as this is now increasingly thought to have toxic effects on cells independent of Aβ. These effects are likely to be mediated by at least two processes, a reduction in CME and an alteration in the endocytosis of endocytic vesicles. Based on our data and on evidence from the literature, we hypothesise that polymorphisms of PICALM could alter the expression of the protein over the lifetime of an individual which would be associated with either an increased risk of developing AD or a protective effect. Further studies are required to elucidate the relative importance of these different roles of PICALM and the relevance of the isoforms to APP processing.

## Methods

### Materials

All chemicals and reagents were purchased from Sigma-Aldrich, Poole, UK, Life Technologies (Invitrogen), Paisley, UK or Fisher Scientific, Leicester, UK and all reactions were performed at room temperature unless otherwise specified. All siRNA (sequences of 21–23 residues) were purchased from Eurofins Genomics, Ebersberg, Germany.

Antibodies were purchased as follows: Anti-APP (clone 22C11, MAB348, Millipore, Watford, UK), anti-β-actin directly conjugated to horseradish peroxidase (HRP, clone AC-15, A3854) or GAPDH (clone GAPDH-71.1, G9295, Sigma-Aldrich), anti-dynamin II (clone 27/Dynamin II, 610263, BD Biosciences, Oxford, UK), anti-clathrin heavy chain (clone 23/Clathrin Heavy Chain, 610499, BD Biosciences), anti-PICALM (NBP1-86658, Novus Biologicals, Cambridge, UK), anti-BACE1 (clone M-83, sc-10748,Santa Cruz Biotechnology, Insight Biotechnology, Wembley UK) and anti-rabbit (PI-1000) and anti-mouse (PI-2000) antibodies conjugated to HRP (Vector Laboratories, Peterborough, UK).

### Cell culture

Neuroglioma H4 cells (ECACC, Porton Down, UK) were cultured in Opti-MEM media containing Glutamax (Invitrogen) supplemented with 4 % foetal bovine serum (FBS, Perbio Science UK Ltd, Cramlington, UK).

### Transfection of cells with siRNAs

The expression of PICALM and clathrin heavy chain (CHC) was depleted using siRNA as previously described [43]. Briefly, H4 cells were seeded in a 6 well plate at a density of 150,000 cells per well 24 h prior to treatment. All procedures were performed in Opti-MEM in the absence of serum. Complexes were pre-formed between the oligonucleotides and oligofectamine (Invitrogen) using 50 pmol oligonucleotide per 4 µl oligofectamine in a total volume of 200 µl Opti-MEM. The complex was subsequently added to cells to give a final oligonucleotide concentration of 50 nM in a total of 1 ml Opti-MEM. Cells were incubated with media, siRNA for green fluorescent protein (GFP) to control for off-target effects of oligofectamine and oligonucleotide, or the siRNA sequences in Table 1 for 4 h. 500 μl of Opti-MEM supplemented with 12 % (v/v) FBS was then added directly to the transfection mixture and cells were then incubated for 48 h and processed as required.

**Table 1:** siRNA sequences used to knock down protein expression

siRNA target	Sequence	References
Total PICALM ‘A’^a^	GCAUACAAUGAAGGAAUUAdTdT	Custom-designed
Total PICALM ‘B’^a^	AAGCCAAGAAUUGCUAUGAdTdT	Custom-designed
PICALM isoform 1	GCAAGUACAUGGGGAGAUCdTdT	Custom-designed
PICALM isoform 2	GUACAUGGGGAGGAUUCACdTdT	Custom-designed
CHC	UAAUCCAAUUCGAAGACCAAUdTdT	[43]
GFP	GGCUACGUCCAGGAGCGCACCdTdT	[43]

^a^The siRNA sequences for total PICALM were directed against regions of the mRNA common to isoforms 1–4

### Analysis of gene expression using quantitative PCR

After siRNA treatment cells were lysed in Trizol™ reagent (Invitrogen) then total RNA was extracted and purified using RQ1 RNase-Free DNase (Promega, Southampton, UK) followed by Qiagen RNeasy™ Mini kits (Qiagen, West Sussex, UK). cDNAs were synthesized from 800 ng RNA with SuperScript^®^ First-Strand Synthesis System (Invitrogen) using Oligo(dT) and Superscript™ II reverse transcriptase. Real-time PCR was carried out on the LightCycler ^®^ 2.0 Real-Time PCR System (Roche Diagnostics GmbH, Mannheim, Germany) using TaqMan gene expression assays (Table 2, Applied Biosystems, Paisley, UK) and LightCycler^®^ TaqMan^®^ Master hot start reaction mix (Roche). Reactions were carried out according to the manufacturer’s protocols. Data were normalised either to polymerase (RNA) II (DNA directed) polypeptide A (POLR2A) or 18S RNA levels. Relative quantification was calculated using the 2^−ΔΔCt^ method [56] using media controls as a reference group to quantify relative changes in target gene expression. Data are presented as fold change in expression normalised to the endogenous reference gene and relative to the untreated cDNA samples.

**Table 2:** TaqMan probes used for real-time quantification of genes following siRNA

Gene	Gene expression assay
Total PICALM	Hs00200318_m1
PICALM isoform 1	Hs00999727_m1
PICALM isoform 2	Hs01003584_m1
PICALM isoform 3	Hs01008128_m1
CHC	Hs00964504_m1
APP	Hs00169098_m1
BACE1 (β-secretase)	Hs01121195_m1
POLR2A	Hs00172187_m1
18S	Hs03928980_g1

All Taqman probes were purchased from Applied Biosystems

### Western blotting

After siRNA treatment, cells were lysed as previously described [57] and total cell protein concentration was determined by bicinchoninic acid protein assay (Pierce, Rockford, IL, USA). Western Blotting was performed using standard methods. Briefly, samples were resolved on 10 % polyacrylamide gels, transferred on to 0.45 µm nitrocellulose membranes (Amersham Biosciences, Little Chalfont, UK), incubated with the relevant primary antibody and detected as previously described [57, 58].

### Internalisation of Alexa-488-labelled transferrin as a measure of CME

This assay was performed according to Al-Soraj and co-workers [43] as described below. siRNA-transfected cells were washed twice with Opti-MEM at room temperature and incubated with Opti-MEM containing 0.2 % (w/v) BSA for 30 min. Cells were then washed twice with Opti-MEM at room temperature and incubated for 0–30 min with Opti-MEM containing 100 nM Alexa488-labelled transferrin (Alexa488-Tf; Life Technologies). Tissue culture plates were immediately placed on ice to inhibit further uptake and washed twice with ice-cold 0.1 M phosphate-buffered saline (PBS) followed by a 1 min incubation in ice-cold acid wash (0.2 M acetic acid, 0.2 M NaCl, pH 2.0) to remove remaining surface label. Cells were then washed three times with PBS at room temperature and trypsinised. This reaction was inhibited with PBS supplemented with 1 % (w/v) BSA, trypsin inhibitor (1 mg/ml) and DNASE (75 µg/ml) before the cell suspension was washed with ice-cold PBS supplemented with 1 % (w/v) BSA and DNASE (75 µg/ml). Cell-associated fluorescence was detected by flow cytometry on a BD FACSCalibur™, BD FACSCanto™ or BD FACSVerse™ analyser (BD Biosciences, Oxford, UK). Cell debris and aggregates were gated out and 10,000 events counted. Data are expressed as fold change over control cells and the mean fluorescent intensity (MFI) is the geometric mean. Data were analysed with Cyflogic 1.2.1 (Cyflo LTD, Turku, Finland) or BD FACSuite™ software.

### Quantification of APP

APP in the lysed cells samples was quantified using the APP DuoSet (R&D Systems, Abingdon, U.K.) following the manufacturer’s guidelines [57, 58]. Briefly, the capture antibody was used at 4 μg/ml in PBS overnight. Plates were blocked with 1 % bovine serum albumin (BSA) and 5 % sucrose in PBS and samples were quantified using a six point standard curve. The biotinylated detection antibody was used at 300 ng/ml and detected using streptavidin-HRP and o-phenylenediamine.

### Quantification of APP fragments: Aβ40, sAPPα, sAPPβ and βCTF

For analysis of Aβ40, media was subjected to immunoprecipitation and ELISA as described previously [57, 58]. Briefly, the ELISA employed the N-terminal Aβ antibody 6E10 (5 mg/ml, Cambridge Bioscience Ltd, Cambridge, UK), as the capture antibody and affinity-purified BAM401AP (0.45 μg/ml, Autogen Bioclear, Calne, UK), specific to the C-terminus of human Aβ40, as the detection antibody. To determine the effect of siRNAs on sAPPα and sAPPβ, media was tested in sandwich ELISAs (IBL, Hamburg, Germany) and intracellular βCTF levels were detected by ELISA (IBL).

### Effect of siRNA to total PICALM on H4 cell viability

Viability studies were performed after incubation with siRNA to total PICALM for 48 and 72 h using the CellTiter 96^®^ MTS Aqueous One Solution Cell Proliferation Assay (Promega) following the manufacturer’s guidelines. The total number of cells was also counted using a Z™ Series Coulter Counter (Beckman Coulter (UK) Ltd, High Wycombe, UK).

### Immunocytochemistry

H4 cells were plated on collagen-coated coverslips as previously described [57] and treated with various siRNA as described above for 48 h. H4 cells were then fixed and processed for immunocytochemistry as previously described [57, 59]. Cells were incubated with primary antibodies in blocking solution (PBS, 3 % serum from the species used to raise the secondary antibody, 1 % bovine serum albumin) overnight at 4 °C. Antibody labelling was detected using appropriate secondary antibodies conjugated to biotin (1:500, Vector Laboratories) followed by avidin-FITC (1:600, Vector Laboratories). Coverslips were mounted on to glass slides using VECTASHIELD^®^ HardSet™ Mounting Medium (Vector Laboratories) with DAPI and visualized using a Leica SP5 confocal laser scanning microscope. GFP siRNA did not affect the localisation or expression of any of the proteins examined compared to media controls so the GFP siRNA images are shown for comparison with those obtained following siRNA for PICALM or clathrin.

### Statistical analysis

Western blots were quantified using Image J, analysed as detailed previously [19] and expressed as relative density to the house keeping protein. Data generated in ELISA assays were quantified by comparing data to standard curves included on each plate using using Graphpad Prism^®^ 5. Results were first normalised to total protein concentration, where relevant and expressed as % of media control values. Both sAPPβ and sAPPα were quantified in ng/ml and then expressed as the ratio of sAPPβ to sAPPα. Where there were only two factors, data were compared to GFP using unpaired Student’s *t* tests, at the two-tailed significance level. Elsewhere data were analysed with one-way ANOVA followed by the relevant post hoc test comparing all data to GFP.

Where necessary, data were transformed to fulfil the assumptions of normality and homoskedasticity and to therefore allow the use of parametric testing. If this proved impossible the equivalent non-parametric test was used.

 All data used to produce Figs. 1, 3, 4, 5, 6, 7, 8 and 9 are shown in the Additional file 1.

## Additional file

10.1186/s12868-016-0288-1 These data were used to produce the graphs shown in Figures 1, 3, 4, 5, 6, 7, 8 and 9.

## Abbreviations

Aβ: β-amyloid
AD: Alzheimer’s disease
Alexa488-Tf: Alexa488-labelled transferrin
APP: amyloid precursor protein
BACE1: β-site APP cleaving enzyme
β-CTF: β-C-terminal fragment
BIN1: bridging integrator 1
CHC: clathrin heavy chain
CME: clathrin-mediated endocytosis
eQTL: expression quantitative trait loci
FBS: foetal bovine serum
GFP: green fluorescent protein
GWAS: Genome Wide Association Study
HRP: horseradish peroxidase
IGAP: International Genomics of Alzheimer’s disease Project
MFI: mean fluorescent intensity
PBS: phosphate-buffered saline
PICALM: phosphatidylinositol binding clathrin assembly protein
Pol II: RNA polymerase II
R-SNAREs: soluble NSF attachment protein receptors
SORL1: sortilin-related receptor
